# The Role of Epigenetic Change in Therapy-Induced Neuroendocrine Prostate Cancer Lineage Plasticity

**DOI:** 10.3389/fendo.2022.926585

**Published:** 2022-07-14

**Authors:** William K. Storck, Allison M. May, Thomas C. Westbrook, Zhi Duan, Colm Morrissey, Joel A. Yates, Joshi J. Alumkal

**Affiliations:** ^1^ Department of Internal Medicine, University of Michigan, Ann Arbor, MI, United States; ^2^ Rogel Cancer Center, University of Michigan, Ann Arbor, MI, United States; ^3^ Department of Urology, University of Michigan, Ann Arbor, MI, United States; ^4^ Department of Urology, University of Washington, Seattle, WA, United States

**Keywords:** Neuroendocrine prostate cancer (NEPC), Lineage plasticity, transdifferentiation, Androgen Receptor (AR), epigenetics

## Abstract

The androgen receptor (AR) signaling pathway is critical for growth and differentiation of prostate cancer cells. For that reason, androgen deprivation therapy with medical or surgical castration is the principal treatment for metastatic prostate cancer. More recently, new potent AR signaling inhibitors (ARSIs) have been developed. These drugs improve survival for men with metastatic castration-resistant prostate cancer (CRPC), the lethal form of the disease. However, ARSI resistance is nearly universal. One recently appreciated resistance mechanism is lineage plasticity or switch from an AR-driven, luminal differentiation program to an alternate differentiation program. Importantly, lineage plasticity appears to be increasing in incidence in the era of new ARSIs, strongly implicating AR suppression in this process. Lineage plasticity and shift from AR-driven tumors occur on a continuum, ranging from AR-expressing tumors with low AR activity to AR-null tumors that have activation of alternate differentiation programs versus the canonical luminal program found in AR-driven tumors. In many cases, AR loss coincides with the activation of a neuronal program, most commonly exemplified as therapy-induced neuroendocrine prostate cancer (t-NEPC). While genetic events clearly contribute to prostate cancer lineage plasticity, it is also clear that epigenetic events—including chromatin modifications and DNA methylation—play a major role. Many epigenetic factors are now targetable with drugs, establishing the importance of clarifying critical epigenetic factors that promote lineage plasticity. Furthermore, epigenetic marks are readily measurable, demonstrating the importance of clarifying which measurements will help to identify tumors that have undergone or are at risk of undergoing lineage plasticity. In this review, we discuss the role of AR pathway loss and activation of a neuronal differentiation program as key contributors to t-NEPC lineage plasticity. We also discuss new epigenetic therapeutic strategies to reverse lineage plasticity, including those that have recently entered clinical trials.

## Introduction

Prostate cancer is the second leading cause of cancer-related death in men in the United States with an estimated 34,500 deaths predicted for 2022 (1). Since the discovery that the majority of prostate cancers respond to androgen depletion (2), androgen deprivation therapy (ADT) has been the principal treatment for metastatic tumors. ADT is also commonly used as adjuvant therapy with surgery or irradiation (3). While most metastatic tumors initially respond to ADT, many tumors will eventually progress to the lethal, castration-resistant form of the disease (4–7). We now know that androgen levels sufficient to activate the androgen receptor (AR) are commonly found in castration-resistant prostate cancer (CRPC) due to intratumoral androgen synthesis or metabolism of adrenally-produced androgen precursors (8). In the past decade, novel AR signaling inhibitors (ARSIs) that inhibit androgen biosynthesis—such as abiraterone acetate (9)—or that competitively bind to the AR and interfere with androgen activation—such as enzalutamide (10), apalutamide (11), and darolutamide (12)—have been tested and approved for the treatment of CRPC.

However, even though ARSIs prolong survival, resistance is nearly universal, and there are limited treatment options once tumors become resistant. Resistance to ARSI treatment may be broken down into two major categories: AR signaling-dependent and AR signaling-independent (13). Maintenance of AR signaling despite continued treatment with ARSIs occurs through multiple mechanisms, including alterations to the AR itself or through compensation by other factors such as the glucocorticoid receptor (14–18).

An AR signaling-independent state is defined by reduced reliance on the AR and activation of other factors, such as MYCN and AURKA that promote cell survival (19). One such AR-independent resistance mechanism is lineage plasticity, wherein tumor cells switch from an AR-driven, luminal differentiation program to an alternate differentiation program (20). Recent work has clarified that lineage plasticity occurs on a continuum and that there are distinct subsets. These subsets include: AR activity-low tumors with decreased AR signaling despite persistent AR expression, amphicrine tumors that have both active AR and neuronal programs in the same cell, double negative tumors that lack AR expression but that do not express a neuronal program, and finally neuroendocrine prostate cancer (NEPC) tumors that lack AR expression, but activate a neuronal program (13).

**Figure 1:**
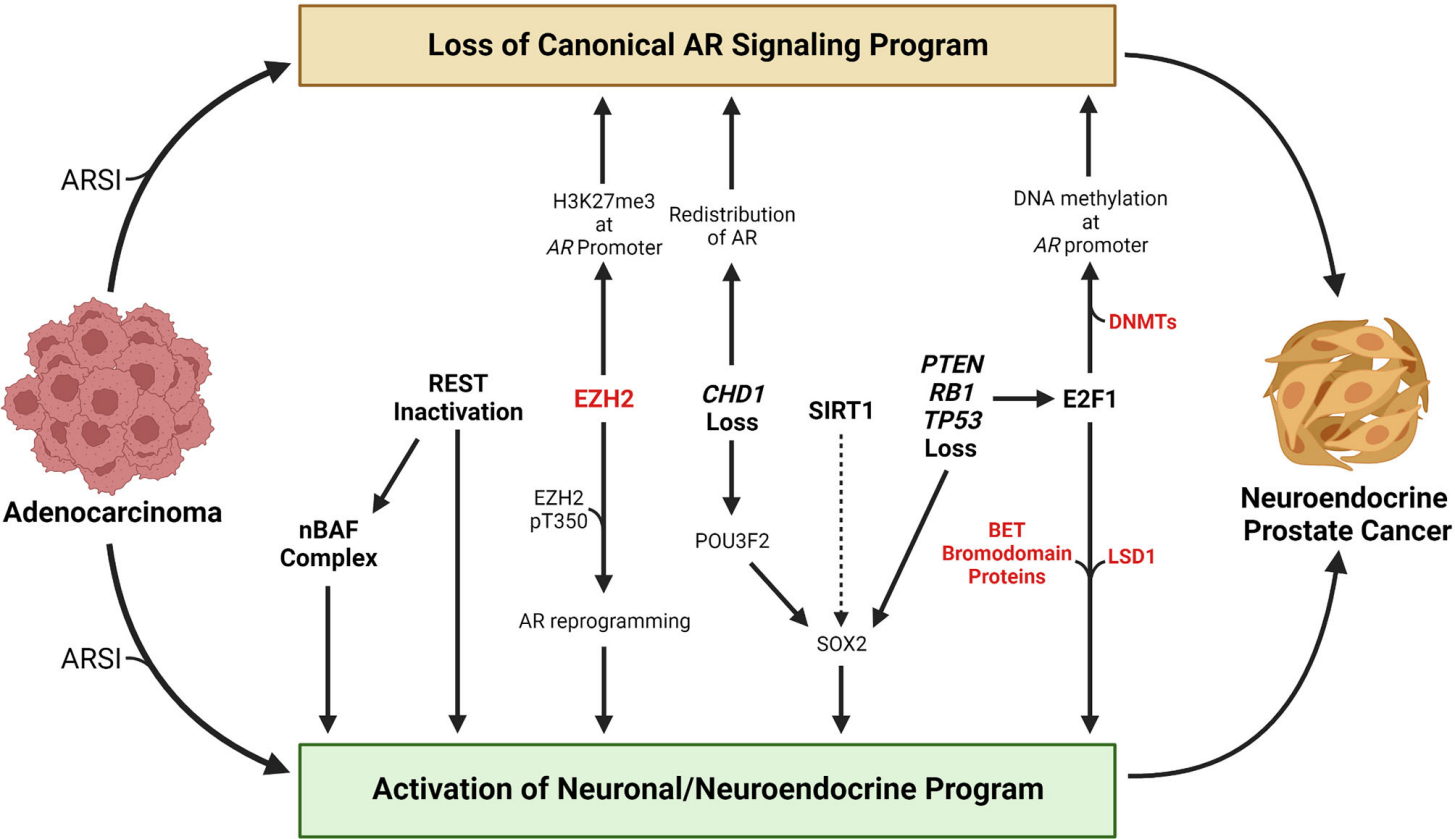
Overview of selected molecular events that contribute to the transition from adenocarcinoma to neuroendocrine prostate cancer. Loss of a canonical AR signaling program and activation of a neuronal or neuroendocrine program are hallmarks of the transition from adenocarcinoma to neuroendocrine prostate cancer. Treatment with AR signaling inhibitors (ARSIs) results in suppression of canonical AR signaling and activation of AR-repressed genes linked with a neuronal or neuroendocrine program in some tumors. Loss of REST’s repressive activity promotes NEPC lineage plasticity, in part through activation of the nBAF chromatin remodeling complex. EZH2 has been shown to silence *AR* expression by catalyzing H3K27me3 at the *AR* promoter. Phosphorylation of the T350 residue on EZH2 results in reprograming of the AR cistrome and activation of stemness or neuronal genes. Loss of the chromatin remodeling factor *CHD1* results in redistribution of the AR away from canonical AR target genes to tumor-specific AR-bound regions. *CHD1* loss also increases chromatin accessibility for the neuronal transcription factor POU3F2 that may contribute to activation of the stemness factor SOX2. The HDAC SIRT1 promotes NEPC lineage plasticity, potentially through activation of SOX2. *PTEN, RB1*, and *TP53* are commonly altered in NEPC, and their loss promotes activation of a neuronal program in part through activation of E2F1 or SOX2. E2F1 has been shown to cooperate with DNA methyltransferases to silence the *AR* through *AR* promoter DNA methylation. E2F1 also cooperates with LSD1 or BET bromodomain chromatin readers to activate a cell cycle or neuronal program. Factors that are currently targetable with drugs that are approved by the Food & Drug Administration or that are in clinical trials are shown in red. Created with BioRender.com.

A key challenge in developing effective treatments for patients with advanced prostate cancer is inter- and even intra-patient tumor heterogeneity (21–25). Therefore, understanding mechanisms by which tumors undergo lineage plasticity may lead to therapeutic approaches to prevent or reverse this virulent form of treatment resistance. In this review, we focus on the NEPC subtype and summarize the epigenetic changes that contribute to NEPC lineage plasticity, discuss methods to identify and classify NEPC, and discuss promising treatment strategies. An overview of selected factors and molecular events discussed in this review that contribute to NEPC lineage plasticity are highlighted in **Figure 1**.

## The Role of AR Pathway Loss in NEPC Lineage Plasticity

It is now well-appreciated that the frequency of tumors with AR pathway loss is increasing in the era of novel ARSIs (26, 27). While *de novo* NEPC comprises less than 1% of newly diagnosed prostate tumors, therapy-induced (t)-NEPC emerging after ARSI treatment represents approximately 20% of CRPC tumors in recent biopsy studies (26, 28). Further, the frequency of NEPC tumors found in a rapid autopsy series appears to be increasing in the era of novel ARSIs (27). This increase in prevalence strongly suggests that ARSIs play a role in the transition of tumors to an NEPC phenotype.

Several studies have confirmed that t-NEPC tumors—like *de novo* NEPC tumors—exhibit significantly lower canonical AR signaling. In a series of metastatic biopsies from men whose tumors were progressing on ARSIs, Aggarwal, et al. performed unsupervised clustering analysis on RNA sequencing data. They identified a distinct cluster termed cluster 2 that was highly enriched for t-NEPC tumors (26). Master regulator analysis demonstrated that the AR was predicted to be the most deactivated transcription factor in cluster 2 tumors compared to the other four clusters. In another report by Beltran, et al., AR mutations—often gain of function events—were commonly found in adenocarcinoma CRPC tumors but were notably absent from t-NEPC tumors (29). These data further suggest that canonical AR signaling becomes dispensable in t-NEPC.

Interestingly, in the report by Aggarwal, et al., approximately 75% of t-NEPC tumors continued to express the AR (26). However, canonical AR signaling was absent or decreased. Importantly, patients were still taking ARSIs at the time of biopsy in many cases, which may have contributed to loss of AR function in the t-NEPC tumors with persistent AR expression. The inverse relationship between canonical AR signaling and activation of an NEPC program suggests that loss of AR function—including through pharmacologic agents—may promote lineage plasticity.

A key function of the AR is to regulate proliferation and differentiation of normal and transformed luminal prostate cells (30). The developing prostate is dependent on androgen signaling, and castration induces involution of the prostate (31–34). *In vitro*, androgen-induced luminal differentiation of prostate epithelial cells is dependent on AR expression (35, 36). Furthermore, tissue recombination experiments using urinary tract epithelia from wild-type and androgen-insensitive mice highlight the importance of the AR for promoting luminal differentiation and the secretory functions of prostatic epithelial cells (37). Previous studies examining the effects of androgen deprivation on prostate cells showed that loss of androgen signaling results in significant apoptosis and dedifferentiation of luminal cells (38–40). Studies using conditional knock out mice deficient for the AR specifically in prostate epithelia revealed that epithelial cell-specific loss of the AR prevents luminal differentiation and leads to loss of glandular in-folding and eventual epithelial sloughing into the prostate lumen (41). These results demonstrate that AR signaling is essential for maintenance of luminal differentiation. Indeed, transcriptional analysis of prostate luminal vs. basal cell populations determined that AR target gene expression is strongly linked with a luminal program, and luminal cells could be distinguished from basal cells based on activation of canonical AR target genes (42, 43). In contrast, the gene expression program of basal cells was activated in aggressive human prostate tumors linked with higher risk of tumor metastasis and shorter survival (43, 44). In our own work, we determined that tumors that exhibit *de novo* resistance to the ARSI enzalutamide have lower AR activity and activation of a basal stemness program, strongly suggesting that AR-independent tumors may be more aggressive (45). These *de novo* resistant tumors also had activation of gene signatures linked to NEPC (45, 46). Overall, these results suggest that in some tumors—perhaps due to the cell of origin (47, 48)—loss of AR signaling may contribute to lineage plasticity, at least in part through loss of a luminal differentiation program.

While the correlation between AR pathway loss and lineage plasticity is clear, recent work sheds new light on how loss of AR signaling—including through ARSIs—might contribute to NEPC lineage plasticity. ADT expands stem-like cells while suppressing non-stem-like cells in tumor samples from patients before and after undergoing androgen deprivation (49). Studies on the highly castration-sensitive patient-derived xenograft (PDX) model BM18 revealed that ADT enriches for a population of prostate cancer cells with both stem-like and luminal characteristics and low AR expression (50). Upon androgen stimulation, these cells proliferate, suggesting that these AR-low cells may promote tumor repopulation after ADT (51).

Recent work by Bishop, et al. demonstrated that the AR also represses activation of alternate differentiation programs, thus helping to maintain luminal identity (52). They developed enzalutamide-resistant cell lines called MR42D and MR42F that continue to express the AR but that have low canonical AR activity (52). These cells harbor a stem-like program with activation of the neuronal transcription factor BRN2 (POU3F2) (53)—a direct target gene repressed by the AR (52). BRN2 was found to activate SOX2, which may contribute to BRN2’s effects on promoting NEPC lineage plasticity (52).

Using these same models, our group recently clarified additional mechanisms that contribute to AR pathway loss-induced NEPC lineage plasticity (54). We determined that enzalutamide treatment of MR42D and MR42F—but not AR-driven parental LNCaP or the CRPC LNCaP derivative line V16D—activates an AR-repressed NEPC lineage plasticity program (54). The chromatin state of MR42D cells appeared to be more conducive to activation of this program, and we determined that the master regulator transcription factor E2F1—in cooperation with the chromatin reader BET bromodomain protein BRD4—appeared to be critical for NEPC reprogramming (54). Using both pre-clinical models and pre-treatment biopsies from a recently completed ZEN-3694 BET bromodomain inhibitor clinical trial (55), we determined that a subset of NEPC tumors harboring high expression of the AR-repressed, E2F1-activated NEPC lineage plasticity program we identified in MR42D cells was strongly linked to prolonged tumor control with BET bromodomain inhibitor treatment. This suggests that these tumors may be particularly susceptible to BET bromodomain inhibition (54). Finally, prior work using LNCaP and LNCaP C-33 cell lines grown in androgen-depleted conditions demonstrated that androgen deprivation is sufficient to induce an NEPC phenotype by inducing RPTPα expression, leading to activation of the MEK/ERK pathway to promote neuroendocrine transdifferentiation (56, 57). Thus, loss of AR function may lead to NEPC lineage plasticity through activation of multiple reprogramming factors.

While activation of AR-repressed genes appears to play an important role in lineage plasticity, recent work by Davies, et al. suggests the AR may also be reprogrammed during the transition to NEPC (58). In that report, they used MR42D and MR42F cells and showed that AR cooperates with the polycomb protein EZH2 in a noncanonical polycomb complex to activate a subset of genes linked to a neuronal program (58). They found that MR42D and MR42F cells have phosphorylation of EZH2 at the T350 residue (58). This post-translational modification is associated with EZH2 activation and activation of NEPC and lineage plasticity-related factors (58). AR and pEZH2-T350 colocalized at non-canonical AR target genes—genes associated with stem cell programs—suggesting that phosphorylation of EZH2 may be associated with redistribution of the AR (58). Importantly, the AR- and pEZH2-T350-bound regions they identified by chromatin immunoprecipitation (ChIP) sequencing were not marked by high levels of the histone methylation mark catalyzed by EZH2—H3K27me3—demonstrating histone methylation-independent mechanisms of EZH2 function (58). Interestingly, EZH2 inhibition reversed the NEPC phenotype, restored canonical AR signaling, and re-sensitized cells to enzalutamide (58). Importantly, the authors also deleted the AR with CRISPR-Cas9, which resulted in further NEPC differentiation (58). These results indicate that even though the AR may be reprogrammed to activate stemness and NEPC genes in cooperation with EZH2, AR’s dominant role in the cells examined may be to repress NEPC differentiation.

## The Role of *PTEN*, *RB1*, and *TP53* Loss in NEPC Lineage Plasticity

Several studies have investigated the genomic alterations associated with NEPC tumors (5, 26, 29). Three important tumor suppressor genes, *PTEN*, *RB1*, and *TP53* are commonly altered in human NEPC vs. AR-dependent prostate cancer (5, 26, 29). Interestingly, these tumor suppressor genes are also known to be important in aggressive neuroendocrine tumors from other organs, including small cell lung cancer (59). Loss of more than one of these tumor suppressor genes is common in NEPC (26, 29).

Pre-clinical studies with cell lines or genetically engineered mouse models have confirmed that loss of *PTEN, RB1*, and *TP53*, are synergistic for reprogramming prostate epithelial cells and promoting NEPC lineage plasticity. Indeed, Mu, et al. demonstrated that combined knockdown of *RB1* and *TP53* in *PTEN*-null LNCaP cells led to lower AR expression, resistance to enzalutamide treatment, and activation of an NEPC program (60). *RB1* and *TP53* loss was linked to increased expression of SOX2, which has also been associated with *RB1* and *TP53* loss in human NEPC (26). SOX2 knockdown abrogated the induction of the lineage plasticity program caused by *RB1*/*TP53* loss and re-sensitized cells to enzalutamide, strongly suggesting that SOX2 was a key downstream effector (60).

Similarly, Ku, et al. developed genetically engineered mouse models to study the impact of *PTEN* loss alone (SKO); *PTEN* and *RB1* loss (DKO); or *PTEN, RB1*, and *TP53* loss (TKO) (61). Compared to SKO, DKO and TKO tumors exhibited lower AR expression and had activation of an NEPC program (61). DKO and TKO tumors were resistant to castration, and mice bearing these tumors had a shorter overall survival vs. SKO mice (61). The authors studied mechanisms that contribute to tumor survival despite surgical castration of mice bearing DKO tumors (recurrent tumors are termed DKO-Cr). Interestingly, they determined that some DKO-Cr tumors acquired *TP53* loss of function mutations (61). This suggests that *TP53* genetic loss may be selected for in *RB1*-deficient tumors after ADT. Like Mu, et al. (60), Ku, et al. determined that DKO and TKO tumors exhibited increased expression of SOX2 (61).

Furthermore, evidence for the importance of *RB1* and *TP53* loss was provided by Park, et al., who sought to identify factors that cooperate to promote NEPC (62). The authors used normal basal prostate epithelial cells and altered the expression or function of several candidate reprogramming factors to create PARCB tumors which contained a dominant negative T **P** 53, constitutively activated myristoylated **A** KT1 (myrAKT1), **R** B1-short hairpin RNA, overexpression of **c**-Myc, and overexpression of **B** CL2 (62). These cells were cultured in an organoid system and then transplanted into immunodeficient mice. The authors then measured tumor growth or NEPC differentiation. To define which factors were necessary to induce NEPC, the authors performed leave-one-out analysis and found that no tumors grew in the absence of c-Myc or myrAKT1 (62). *RB1* and *TP53* loss—together or individually—were indispensable for NEPC (62). This further demonstrates that loss of *RB1* and inactivation of *TP53* are important factors in the conversion of cells to NEPC.

Despite a very clear association between *PTEN, RB1*, and *TP53* loss and the NEPC phenotype, these genomic changes alone are insufficient to promote lineage plasticity in patient tumors. Indeed, many NEPC tumors do not exhibit these genomic alterations, and some non-NEPC CRPC tumors harbor genomic alterations in these genes (29, 63). For example, Beltran, et al. determined that only 70% of NEPC tumors harbored *RB1* loss vs. 32% of CRPC. Sixty-seven percent of NEPC tumors harbored *TP53* mutations vs. 31% of CRPC (29). Nyquist, et al. profiled 410 metastatic biopsies from patients with CRPC and found that 40% of those harboring combined *RB1*/*TP53* loss were adenocarcinomas (63). Furthermore, Nyquist, et al. generated combined *RB1*/*TP53* knockouts in *PTEN*-deficient LNCaP cells using CRISPR-Cas9. While AR transcriptional activity was lower in the knockout cells vs. parental cells, NEPC gene expression was not increased (63). This suggests that other reprogramming factors or the tumor microenvironment that was absent from these *in vitro*-generated cell lines may be important to induce NEPC. Finally, given the importance of RB1 in restraining proliferation, it is also possible that *RB1* loss in a subset of tumor cells that had already undergone NEPC lineage plasticity from an adenocarcinoma phenotype may provide a proliferative advantage, accelerating the shift in the tumor population to an NEPC phenotype.

## The Role of Epigenetic Change in NEPC Lineage Plasticity

Gene expression is controlled not only by DNA sequence alterations, but also by epigenetic mechanisms such as DNA methylation, histone modifications, and chromatin accessibility. There is a wealth of data demonstrating the importance of epigenetic change for NEPC lineage plasticity. Indeed, loss of AR signaling in prostate cancer lineage plasticity is strongly associated with extensive epigenetic reprograming (64). Distinct DNA methylation and histone modification profiles have been shown to be correlated with tumor progression (65–68), and various epigenetic factors have been shown to induce stemness or epithelial-mesenchymal transition (EMT) (61, 69, 70).

### DNA Methylation

DNA methylation is an important epigenetic mark wherein a methyl group is covalently attached to the 5’ carbon of the pyrimidine rings on cytosines, and these methylation patterns are retained after cell division (71). Maintenance DNA methylation is catalyzed by the DNA methyltransferase DNMT1 that prefers hemimethylated DNA as its substrate. DNMT3A and DNMT3B are capable of methylating both hemimethylated and non-methylated DNA and are referred to as *de novo* methyltransferases (71).

Expression of DNMT1 is increased in prostate epithelial cells with loss of *RB1* (72). Mechanistically, RB1 negatively regulates the transcription factor E2F1, and E2F1 can activate *DNMT1* expression through interactions with the *DNMT1* promoter (72). Accordingly, overexpression of E2F1 increases *DNMT1* expression (72). E2F1 overexpression was sufficient to repress expression of the *AR* and *AR* promoter-driven reporter constructs while *DNMT1* knockdown reactivated *AR* expression in AR-negative human primary prostate epithelial cells (73). These results suggest that DNMT1 is an important mediator of E2F1-induced *AR* silencing.

*In vivo* studies utilizing the murine prostate cancer model TRAMP (74) demonstrated the potential of DNA methyltransferase inhibition for the treatment of prostate cancer and AR reactivation (75, 76). Upon puberty, the TRAMP model expresses SV40 large T antigen specifically in prostate epithelial cells. SV40 large T antigen blocks RB1 and TP53 function and leads to the development of poorly differentiated tumors (74). Recent work suggests that the poorly-differentiated tumor cells that emerge may arise from pre-existing malignantly transformed progenitor cells, rather than adenocarcinoma cells that undergo transdifferentiation (77).

TRAMP mice exhibit elevated E2F1 and DNMT1 levels in premalignant and malignant prostate cancer lesions (75). Treatment with the DNA demethylating agent 5-aza-2’-deoxycytidine (5-aza) reactivated AR expression, prevented the development of poorly differentiated prostate cancer and lymph node metastases, and significantly extended survival vs. control-treated mice (75). 5-aza treatment also caused tumor regression in TRAMP mice with established tumors (76). Finally, treatment with 5-aza with or without castration in TRAMP mice demonstrated that combined 5-aza + castration treatment had the lowest number of poorly differentiated prostate cancer tumors and metastases, suggesting combination treatment is a promising strategy to reverse lineage plasticity and castration-resistance (76).

DNA methylation has been also implicated in regulating AR expression in AR-null cell line models and patient tumor samples (78–80). Treatment with 5-aza led to re-expression of the *AR* with concomitant DNA demethylation of the *AR* promoter in the AR-negative DuPro, TSU-PR1 and DU145 cell lines (78, 80). Thus, DNMT inhibitors may be a promising class of drugs to restore AR expression in subsets of AR-null prostate cancer.

Genome-wide patterns of DNA methylation can also be used to distinguish between benign prostate tissue, primary prostate cancer, and metastatic CRPC (68). Using differential DNA methylation profiling, Zhao, et al. was able to classify t-NEPC patient tumor samples (68). In an analysis of AR+, amphicrine, double negative, and NEPC PDXs, Brennen, et al. determined that loss of *AR* expression was correlated with *AR* promoter hypermethylation specifically in NEPC models (81). Beltran, et al. examined DNA methylation in NEPC vs. adenocarcinoma tumors and found differences in pathways linked to cell-cell adhesion, EMT, and stem cell programs (29). Another study by Beltran, et al. examined DNA methylation in cell-free DNA (cfDNA) samples from patients with t-NEPC or adenocarcinoma to determine unique features found in NEPC (82). Hypermethylation of the *ASXL3* and *SPDEF* genes was observed in the NEPC samples (82). Additionally, NEPC tumors exhibited hypomethylation of both the NEPC marker *INSM1* and the plasticity gene *CDH2* (82). Altogether, these studies suggest that DNA methylation may play an important role in regulating expression of the *AR* and genes linked to lineage plasticity.

### Histone Methyltransferases, Demethylases, and Deacetylases

Histones possess unstructured N-terminal tails that are subject to post-translational modifications that influence gene expression. Among the most well-studied are lysine methylation and acetylation that alter chromatin organization and accessibility (83). Two classes of histone writers—histone methyltransferases and histone acetyltransferases that methylate or acetylate specific lysines, respectively—are responsible for catalyzing the addition of these marks. Histone methylation and acetylation are tightly regulated processes and are reversible through the activity of histone erasers—histone demethylases and histone deacetylases (HDACs). Finally, chromatin readers are a third major class of epigenetic factors that are thought to “read,” or interpret, histone modifications by binding these marks and recruiting transcription factors and other regulatory proteins to chromatin.

Histone methylation can either activate or repress gene transcription depending on the specific lysine residue that is modified, and repressive histone methylation has been linked to *AR* expression loss (84). Using AR- and AR+ NEPC PDX models, Kleb, et al. demonstrated that the *AR* promoter was enriched with the repressive histone modifications H3K27me3 and H3K9me2 in *AR*-null tumors (84). EZH2, the histone methyltransferase responsible for the H3K27me3 mark, has been shown to be upregulated in NEPC (29, 69, 85, 86). Ku, et al. showed that EZH2 catalytic inhibition in DKO and TKO tumors reactivated AR signaling and re-sensitized tumors to enzalutamide while simultaneously decreasing expression of NEPC target genes (61). These data support the importance of histone methylation mediated by EZH2 for *AR* repression in subsets of NEPC.

In addition to histone methyltransferases, histone demethylases may also play a role in CRPC. Lysine specific demethylase 1 (LSD1/KDM1A) is known to demethylate lysine 4 on histone H3 and lysine residues on several non-histone proteins, including TP53, E2F1, DNMT1, and HIF-1α (87). LSD1 may also indirectly demethylate lysine 9 on histone H3, including at canonical AR target genes (87) or cell cycle genes (88). Han, et al. demonstrated the importance of LSD1 for E2F1 chromatin binding in *RB1*-deficient CRPC C4-2 and VCaP cell lines (89). Collectively, these data suggest that *RB1* inactivation may confer vulnerability to LSD1 inhibition.

Recent work from our group demonstrates that LSD1 promotes AR-independent survival of CRPC cells independently of its catalytic function (90). We also recently identified a neuronal-specific isoform of *LSD1* called *LSD1+8a* that was specifically expressed in NEPC vs. adenocarcinoma in PDX models and metastatic biopsies (91). The splicing factor SRRM4 that is overexpressed in NEPC was shown to mediate the alternative splicing of *LSD1+8a* (91). Through gain of function studies, LSD1+8a and SRRM4 were shown to co-regulate a distinct set of genes from canonical LSD1 (91). SRRM3 has also been shown to mediate alternative splicing of *LSD1+8a* (92). Collectively, these data suggest *LSD1* splice variants such as *LSD1+8a* may be biomarkers for NEPC or contribute to NEPC lineage plasticity.

HDACs lead to changes in chromatin accessibility and are important in AR signaling and prostate cancer (93–96). Ruan, et al. demonstrated that the HDAC SIRT1 is upregulated in NEPC and showed that overexpressing SIRT1 promotes NEPC lineage plasticity (94). SIRT1 has been shown to promote upregulation of SOX2 in breast cancer (97), liver cancer stem cells (98), and bone marrow-derived mesenchymal stem cells (99). Thus, it is possible that SOX2 upregulation by SIRT1 may also contribute to NEPC lineage plasticity. In summary, although the precise mechanisms by which HDACs promote NEPC remain unclear, these data suggest that HDACs may be important therapeutic targets in this disease.

### Histone Acetylation, Chromatin Accessibility, and Chromatin Readers

The accessibility, or openness, of chromatin is an essential determinant of gene expression. Histone acetylation reduces the positive charge on histones and is thought to de-compact chromatin by weakening histone binding to negatively charged DNA (83). Histone acetylation is also recognized by bromodomain proteins that recruit transcriptional machinery (100). Chromatin accessibility is regulated by chromatin remodelers, which regulate transcription by controlling the positioning of nucleosomes, the basic repeating unit of eukaryotic chromatin (101). Recent studies show that ARSI treatment may induce widespread changes in chromatin accessibility that may contribute to lineage plasticity (54, 102–104).

Pomerantz, et al. evaluated the epigenomes of human prostate samples and PDXs (103). They identified reprogramming of the AR cistrome between benign prostate tissues, hormone sensitive prostate cancer tissues, and CRPC tissues, identifying over 17,000 AR binding sites and over 16,000 H3K27ac sites enriched in CRPC (103). Assay for Transposase Accessible Chromatin (ATAC)-sequencing in both normal and primary tumor specimens demonstrated chromatin accessibility at the AR binding sites, and the DNA was hypomethylated in these regions (103). In metastatic CRPC, AR was reprogrammed to sites associated with developmental prostate programs (103). These data demonstrate that chromatin accessibility and the AR cistrome change with prostate cancer progression, contributing to reactivation of prostate developmental pathways in CRPC cells (103). While this study did not specifically examine NEPC, it is quite possible that reactivation of a more primitive developmental program may facilitate eventual commitment to non-luminal lineages.

A well-studied chromatin remodeler in prostate cancer is CHD1. Deletion of *CHD1* in normal prostate cells altered the chromatin landscape and led to AR redistribution from lineage commitment regions to tumor-specific AR-bound regions (105). Similarly, *CHD1* loss resulted in enzalutamide resistance by promoting neuronal differentiation (104). *CHD1* loss increased chromatin accessibility for four factors associated with activation of non-luminal lineage programs: NR3C1, POU3F2, TBX2, and NR2F1 (104). Importantly, deletion of each factor by CRISPR-Cas9 re-sensitized *CHD1* knockout cells to enzalutamide (104).

The restrictive element-1 silencing transcription factor (REST) is a repressor of neuronal differentiation (106). REST has been shown to cooperate with the AR to repress neuronal gene expression (107). Downregulation of REST resulting from hypoxic conditions induces neuroendocrine differentiation in CRPC (108), and REST knockdown increases stemness and EMT gene expression in NEPC models (109). Loss of REST’s repressor activity through a splicing-in event by SRRM3/4 promotes BAF53B (*ACTL6B*) expression in both amphicrine and NEPC tumors (13). BAF53B is a component of the neuron-specific nBAF chromatin remodeling complex that regulates gene expression and differentiation (110). The exchange of BAF53A and BAF45A subunits within the BAF complex for homologous BAF53B and BAF45B subunits within neuron-specific BAF (nBAF) complexes promotes a chromatin switch to a differentiated neuronal phenotype in post-mitotic neurons (111–113). Cytra, et al. demonstrated that BAF53B and BAF45B are highly expressed in NEPC but absent from benign prostate, localized prostate cancer, or CRPC adenocarcinoma samples, demonstrating high specificity for the neuroendocrine phenotype (102). Interestingly, neither BAF53B nor BAF45B knockdown had an effect on NEPC cell proliferation. Therefore, the authors suggest that BAF53B and BAF45B expression may be specific for the NEPC phenotype, but not a critical mediator of NEPC aggressiveness (102). Finally, BET bromodomain proteins—such as BRD4 that recognizes the H3K27ac mark—have been shown to play an important role in NEPC. Our prior work demonstrated that BRD4 cooperates with E2F1 to drive lineage plasticity (54). We found strong colocalization of H3K27ac and BRD4 signals in t-NEPC cell lines (54). Furthermore, BET bromodomain inhibition abrogated E2F1 induction of NEPC lineage plasticity genes and suppressed growth of E2F1-high t-NEPC cell lines, strongly suggesting these factors cooperate (54). Collectively, these studies demonstrate that epigenetic factors play a key role in promoting NEPC lineage plasticity. Because of the diversity of epigenetic regulators that are altered in NEPC, it may be necessary to target multiple epigenetic regulators simultaneously to reverse or prevent lineage plasticity.

## Identifying NEPC Tumors

Currently, several approaches are used to identify tumors that have undergone NEPC lineage plasticity. Histology and examination of morphologic features are the gold standard to distinguish *de novo* NEPC from adenocarcinoma (114). However, use of morphology alone is challenging in tumor specimens from patients who have been treated with agents such as ADT or ARSIs that may alter cellular morphology and give a false impression of the phenotype of a tumor. Another approach is to use immunohistochemistry for canonical NEPC markers, including CHGA, SYP, NSE, CD56 (NCAM1), or INSM1 that are highly expressed in *de novo* NEPC (114–116). However, many t-NEPC tumors do not have high expression of these genes, presumably because they are not as far along the continuum of NEPC differentiation as a *de novo* NEPC tumor (26).

Another approach to determine the program of a cell more accurately is through simultaneous measurement of multiple markers through RNA-sequencing. Indeed, we now know that lineage plasticity exists on a continuum (13). As previously discussed, Labrecque, et al. used rapid autopsy samples and identified five tumor subtypes based on IHC and gene expression that may represent a more accurate classification system than current standard pathologic assessment (13). Other studies have created gene expression signatures associated with NEPC or t-NEPC using clinical cohorts (26, 29). These studies had high levels of cross validation and accuracy when applied to external cohorts and further suggest molecular classification of tumors through gene expression may be preferable. However, the development of assays that are easy to use and interpret will be critical before transcriptional profiling is routinely used for molecular subtyping in clinical practice.

Though only a minority of patients will eventually develop t-NEPC after ARSI treatment, patients with t-NEPC are often not identified because metastatic biopsies are not routinely done in practice and assessment for an NEPC phenotype remains challenging. NEPC detection is a key area that must be improved upon—both to identify men for whom conventional therapies such as ARSIs will not be effective and to identify patients who might be eligible for NEPC-focused clinical trials. To date, clinical trials in NEPC have generally used histologic assessments, clinical phenotype (e.g., liver metastasis in absence of PSA progression), and blood markers (e.g., serum or tissue CGA) as enrollment criteria. Several other therapeutic trials have focused on specific clinical markers to enrich for patients with “aggressive variant prostate cancer” (AVPC), though this clinical classification likely includes a mixture of tumor subtypes that may or may not be AR-driven.

Some phase II studies (NCT04592237, NCT03263650) enrolling AVPC used mutations or loss of function events in the tumor suppressors *PTEN, RB1*, and *TP53*. Given the key role these genes play in both suppressing lineage plasticity and proliferation (60, 61), enriching clinical trials for patients whose tumors harbor loss of these genes is a pragmatic approach to test treatments predicted to be active in highly proliferative tumors, including NEPC. Detection of genomic loss in these genes is relatively straightforward vs. RNA-based assays and may enrich for NEPC tumors. However, loss of these genes is clearly not specific for NEPC (60–63, 117).

There remains an unmet need to identify tumors that have undergone lineage plasticity *via* non-invasive methods. Molecular imaging may be one promising avenue. Radionuclide scans using tracers conjugated to prostate specific membrane antigen (PSMA) and dihydrotestosterone (DHT) have been widely studied in prostate cancer (118). PSMA or DHT low/negative tumors that are metabolically active — FDG or choline positive — are enriched for aggressive tumors (119, 120) or those that are AR-independent, such as NEPC (121). DLL3 has recently gained recognition as an NEPC marker and drug target (122). The authors of a recent study developed a PET imaging method using a (^89^Zr)-labeled DLL3 targeting antibody that specifically detected neuroendocrine PDXs *in vivo* (123).

Promising strategies for identification of NEPC non-invasively *via* circulating blood markers have also been identified. Beltran, et al. published a proof of concept study in which they identified NEPC circulating tumor cells (CTCs) *via* morphology and immunofluorescence staining (124). A later study found that detection of an NEPC phenotype in CTCs was associated with significantly worse overall survival after starting an ARSI (125). Another blood-based method is cfDNA assays. One recent study detected NEPC features *via* whole exome sequencing and whole genome bisulfite sequencing (82). An NEPC score was created using a targeted panel of key DNA genomic alterations and 20 hyper- or hypomethylated sites (82). Another study created a NEPC signature from tissue samples using methylated DNA immunoprecipitation and sequencing (MeDIP-seq) that was subsequently used to predict the presence of NEPC *via* cfDNA (126). The optimal cutoff produced results with 100% sensitivity and 95% specificity in a validation cohort. The clear implication from these studies is that non-invasive identification of NEPC may soon become a reality in patients with tumor burden significant enough to lead to detectable CTCs.

## Clinical Trials in NEPC

Aggarwal, et al. determined that patients with small cell histologic features or the cluster 2 transcriptional program in their CRPC biopsies were associated with poor overall survival (26). Thus, t-NEPC tumors appear to be more aggressive than adenocarcinoma CRPC tumors. The standard of care for both *de novo* and t-NEPC is chemotherapy based on the treatment regimens used in small cell lung cancer (127–132). Most commonly, platinum doublets (e.g., cisplatin or carboplatin) with etoposide or a taxane (e.g., docetaxel or cabazitaxel) are utilized. High initial rates of response were also found to docetaxel and carboplatin in the clinically-defined AVPC subset that included some NEPC tumors (130). While most patients respond, relapse is universal with a median survival of 1-2 years from the time of diagnosis (128, 130). Though a trial of the single agent anti-PD1 inhibitor avelumab showed limited efficacy in men with NEPC (NCT03179410) (133) we await the results of trials incorporating checkpoint inhibitors with standard platinum doublets — as is now standard of care in small cell lung cancer.

There are few published reports of clinical trials using targeted agents focused on NEPC patients, specifically. One of the earliest phase II trials tested the aurora kinase inhibitor alisertib (NCT01799278) (134). MYCN is a known regulator of lineage plasticity that is upregulated in subsets of NEPC (135), and AURKA appears to be important for stabilizing MYCN (136). Therefore, these investigators tested alisertib in patients with NEPC. Of note, MYCN or AURKA upregulation was not required for enrollment. This trial did not meet its primary endpoint of 6-month progression free survival. However, the subpopulation of patients with increased AURKA expression — 16% of study population — did appear to have longer overall survival (134).

Another targeted agent, rovalpituzumab tesirine (an antibody drug conjugate against DLL3), was tested in a phase I/II trial (NCT02709889) that included 18 patients with NEPC and found an objective response rate of 10% (137). There are several ongoing targeted trials specifically enrolling patients with NEPC or AVPC with targeted agents summarized in **Table 1**.

**Table 1:** Recent clinical trials in neuroendocrine prostate cancer.

Drug	Type or target	Combination agent	Phase	Indication	Trial identifier	Status
**Targeted**						
MLN8237 (Alisertib)	Aurora kinase A	N/A	2	NEPC	NCT01799278	Completed
Rovalpituzumab tesirine	DLL3 (Antibody Drug Conjugate)	N/A	1/2	NEPC	NCT02709889	Completed
Olaparib	PARP	Cabazitaxel/carboplatin	2	AVPC	NCT03263650	Active, not recruiting
BXCL701 (Talabostat)	DPP (DPP8, DPP9)	Pembrolizumab	1b/2	CRPC, NEPC	NCT03910660	Recruiting
Niraparib	PARP	Carboplatin/cabazitaxel +/- cetrelimab	2	AVPC	NCT04592237	Recruiting
Levatinib	VEGFR	Pembrolizumab	2	NEPC	NCT04848337	Recruiting
AMG 757 (Tarlatamab)	DLL3 (Bispecific T cell Engager)	N/A	1	NEPC	NCT04702737	Recruiting
BXCL701 (Talabostat)	DPP (DPP8, DPP9)	Cetrelimab	2	CRPC, t-NEPC	NCT04926181	Not yet recruiting
**Epigenetic**						
DS-3201	EZH1/2	Ipilimumab	1	AVPC (*TP53, RB1, PTEN* loss)	NCT04388852	Recruiting
ZEN-3694	BET	Enzalutamide/Pembrolizumab	2	t-NEPC (also includes CRPC arm)	NCT04471974	Recruiting
ZEN-3694	BET	Enzalutamide	2b	CRPC (focused primarily on those with poor response to prior abiraterone)	NCT04986423	Recruiting
**Chemo/immunotherapy**						
Avelumab	Immunotherapy (anti-PD-L1)	N/A	2	NEPC, AVPC	NCT03179410	Completed
Nivolumab, Ipilimumab	Immunotherapy (anti-PD1, CTLA-4)	Carboplatin, cabazitaxel	2	NEPC, AVPC	NCT04709276	Recruiting
Pembrolizumab	Immunotherapy (anti-PD-L1)	Platinum doublet	1	NEPC, other GU malignancies	NCT03582475	Recruiting

N/A, Not Applicable.

Multiple phase I-II studies have tested epigenetic targeted agents in CRPC. The majority of completed and ongoing trials of epigenetic targeted agents recruited from the general CRPC population and were not specifically focused on NEPC, thus making it difficult to determine the effectiveness of these agents in this molecular subset.

As stated previously, there are marked DNA methylation differences between adenocarcinoma and NEPC tumors, and DNMTs have been implicated as key lineage plasticity factors (29). While DNMT inhibition showed promise in preclinical models of NEPC (75, 76), clinical trials of DNMT inhibitors in general CRPC populations (NCT00384839) have reported a low proportion of responders (138).

HDAC regulate AR activity (139) and have been found to be upregulated in NEPC cell lines (94). HDAC inhibitors are among the best studied epigenetic therapies in prostate cancer in both preclinical (95) and clinical trials (93, 140–142). In the CRPC population, single agent activity has been low, but combination therapy with the ARSI casodex appeared promising (140).

As previously discussed, BET bromodomain proteins are chromatin readers that cooperate with several transcription factors, including the AR and E2F1 (54, 143). Several clinical trials with BETi have been conducted in prostate cancer, though none was specifically focused on NEPC (55, 144, 145). In a recent study with the BETi ZEN-3694, pre-treatment biopsies and clinical factors were examined to identify markers of response (NCT02711956) (55). In an exploratory analysis, shorter time on ARSI treatment prior to study entry was linked to better chance of response to ZEN-3694 (55). Further, examination of RNA-sequencing from baseline, pre-treatment biopsies demonstrated that lower canonical AR transcriptional activity was associated with longer time to progression while on treatment (19 vs. 45 weeks) (55). Four patients enrolled on this trial exhibited an NEPC program at baseline (54). Of those, two were long-term responders and had high expression of BRD4 and E2F1 and activation of an AR-repressed, E2F1-activated NEPC lineage plasticity program vs. the other two patients whose tumors progressed more rapidly. This suggests that E2F1/BRD4-activity-high NEPC tumors may be particularly susceptible to BET bromodomain inhibition. ZEN-3694 is currently being tested in combination with enzalutamide + pembrolizumab in a phase II trial that includes a t-NEPC cohort (NCT04471974). A separate study of ZEN-3694 in combination with enzalutamide vs. enzalutamide alone is recruiting a cohort that had poor response to abiraterone (NCT04986423)—based on results from the prior phase I ZEN-3694 trial (55)—with the hope that doing so will enrich for tumors that have undergone lineage plasticity.

Other epigenetic targets are also being assessed in ongoing trials. The histone demethylase LSD1 is being studied in a phase I/II study that includes neuroendocrine tumors (NCT02712905). Inhibitors of EZH2 are also being tested in ongoing trials alone or in combination with ARSIs (NCT04986423). Notably, a phase I trial of DS3201 (EZH1/2 inhibitor) and ipilimumab is specifically recruiting AVPC in addition to other genitourinary cancers (NCT04388852).

## Identifying Patients at Risk for Therapy-Induced NEPC Lineage Plasticity

The current focus of t-NEPC research is how to identify these tumors more accurately and treat them more effectively once they develop after ARSI treatment. However, identification of tumors at greatest risk of ARSI-induced AR pathway loss and t-NEPC lineage plasticity before these changes occur may lead to better outcomes. Indeed, recent reports indicate that significant heterogeneity exists in tumors that have undergone lineage plasticity (13), which may contribute to resistance to NEPC-directed therapies. Thus, it may be preferable to identify tumors before they have undergone ARSI-induced NEPC lineage plasticity.

Understanding lineage plasticity risk requires a longitudinal assessment of patients with matched assays (before treatment and at the time of progression). We do not yet know the best measures of risk of NEPC lineage plasticity, and there are no markers that exist to risk-stratify patients. Therefore, we believe it will be important to measure several factors that may contribute to NEPC lineage plasticity risk: genomic, epigenomic, and microenvironmental features. Whole genome or whole exome DNA sequencing can identify genomic loss of known tumor suppressor genes (e.g., *PTEN, RB1*, and *TP53*). Single cell assays such as ATAC sequencing paired with RNA sequencing may provide information about the baseline gene expression program of distinct cell populations and their capacity to turn on other gene expression programs, including those linked with NEPC lineage plasticity. Spatial profiling may help to unravel immune and microenvironmental contributions to lineage plasticity and will be crucial for understanding the communication and cooperativity of different cell populations present in a tumor. Inclusion of non-invasive assays using CTCs or cfDNA may help facilitate serial collection of samples that may give clues about risk of NEPC lineage plasticity that do not require invasive, repeated sampling of patients.

Provided that the correct samples and analytes are collected at baseline, such studies to determine NEPC lineage plasticity risk could be prospective or retrospective. Our group has recently initiated a prospective study called the MIchigan ONCOlogy Multi-omic Assessment of Tumor Change and Heterogeneity (Mi-ONCOMATCH) that incorporates many of the aforementioned assays in an effort to improve our ability to identify those at greatest risk of developing t-NEPC after ARSI treatment. Once studies establishing markers of lineage plasticity risk are completed, prospective validation will be necessary. It is our hope that improvements in non-invasive detection through blood-based assays or molecular imaging will hasten completion of such studies and improve our ability to identify those at greatest risk of developing t-NEPC. The next critical step will be to develop clinical trials testing drugs that suppress induction of an NEPC program or that maintain AR expression and signaling. We believe that epigenetic therapies may be a particularly promising approach for these patients.

## Conclusion

Both genetic and epigenetic changes play a key role in therapy-induced AR pathway loss and t-NEPC lineage plasticity. Understanding mechanisms by which t-NEPC lineage plasticity occurs and markers that indicate this transition has taken place are critical for making progress for this group of patients. We predict that combination clinical trials with ARSIs + lineage plasticity-modifying epigenetic therapies alone or with other targeted agents will be critical for making progress. Advances in detection of lineage plasticity risk will be important to prevent this newly appreciated, aggressive form of ARSI resistance from happening in the first place.

## Author Contributions

WKS, AMM, TCW, and JJA conceived the article. WKS, AMM, TCW, CM, and JJA reviewed the literature and wrote the first draft of the manuscript. WKS made the figure. TCW made the table. WKS, ZD, CM, JAY, and JJA edited the manuscript.

## Funding

This work was supported by National Cancer Institute (NCI) R01 (CA251245); The Pacific Northwest Prostate Cancer Specialized Programs of Research Excellence (SPORE) NCI P50 CA097186; the Michigan Prostate SPORE NCI P50 CA186786; the NCI Drug Resistance and Sensitivity Network NCI P50 CA186786-07S1; NCI Training Grant T32 CA009676; NCI Training Grant 5-T32 CA180984-07; NCI Training Grant T32 CA009357; Department of Defense Idea Award (W81XWH-20-1-0405); National Comprehensive Cancer Network (NCCN)/Astellas Pharma Global Development Award; a Prostate Cancer Foundation Challenge Award; and a Sheppard Family Foundation Sheppard Scholar Award.

## Conflict of Interest

JJA reports consulting and speaker’s fees from Astellas Pharma, consulting fees from Dendreon, consulting fees from Merck, consulting fees from Bristol Myers Squibb, and research support to his institution from Astellas Pharma, Zenith Epigenetics, and Beactica.

The remaining authors declare that the research was conducted in the absence of any commercial or financial relationships that could be construed as a potential conflict of interest.

## Publisher’s Note

All claims expressed in this article are solely those of the authors and do not necessarily represent those of their affiliated organizations, or those of the publisher, the editors and the reviewers. Any product that may be evaluated in this article, or claim that may be made by its manufacturer, is not guaranteed or endorsed by the publisher.
